# Distinct changes in brain metabolism in patients with dementia and hearing loss

**DOI:** 10.1002/brb3.3374

**Published:** 2024-01-06

**Authors:** Ji Hyuk Han, Sangwon Lee, Seong Hoon Bae, Mijin Yun, Byung Seok Ye, Jinsei Jung

**Affiliations:** ^1^ Department of Otorhinolaryngology Yonsei University College of Medicine Seoul Republic of Korea; ^2^ Department of Nuclear Medicine Yonsei University College of Medicine Seoul Republic of Korea; ^3^ Department of Neurology Yonsei University College of Medicine Seoul Republic of Korea; ^4^ Graduate School of Medical Science Yonsei University College of Medicine Seoul Republic of Korea; ^5^ Brain Korea 21 Project Yonsei University College of Medicine Seoul Republic of Korea

**Keywords:** brain metabolism, cognitive impairment, dementia, FDG‐PET, hearing loss

## Abstract

**Introduction:**

Previous studies have reported that hearing loss (HL) is associated with dementia, although the mechanistic underpinnings remain elusive. This study aimed to evaluate the changes in brain metabolism in patients with HL and different types of dementia.

**Methods:**

Patients with cognitive impairment (CI) and HL treated at the university‐based memory clinic from May 2016 to October 2021 were included. In total, 108 patients with CI and HL prospectively underwent audiometry, neuropsychological test, magnetic resonance imaging, and ^18^F‐fluorodeoxyglucose positron emission tomography. Twenty‐seven individuals without cognitive impairment and hearing loss were enrolled as a control group. Multivariable regression was performed to evaluate brain regions correlated with each pathology type after adjusting for confounding factors.

**Results:**

Multivariable regression analyses revealed that Alzheimer's disease‐related CI (ADCI) was associated with hypometabolic changes in the right superior temporal gyrus (STG), right middle temporal gyrus (MTG), and bilateral medial temporal lobe. Lewy body disease‐related CI (LBDCI) and vascular CI were associated with hypermetabolic and hypometabolic changes in the ascending auditory pathway, respectively. In the pure ADCI group, the degree of HL was positively associated with abnormal increase of brain metabolism in the right MTG, whereas it was negatively associated with decreased brain metabolism in the right STG in the pure LBDCI group.

**Conclusion:**

Each dementia type is associated with distinct changes in brain metabolism in patients with HL.

## 1 INTRODUCTION

Epidemiologic studies have reported a high prevalence of dementia in adults with hearing loss (HL), suggesting that HL may be a risk factor for dementia (F. R. Lin et al., 2011; Livingston et al., 2020). However, the relationship between HL and dementia remains unclear. Several mechanisms, such as common pathology, auditory input deprivation, and increased cognitive load, have been proposed to explain the relationship between peripheral HL and cognitive decline (Griffiths et al., 2020), although these remain controversial and poorly understood.

Several types of dementia with distinct pathophysiology exist, including Alzheimer's disease (AD), vascular pathology, Lewy body‐related disease (LBD), and mixed‐type pathologies (Rahimi & Kovacs, 2014). AD and LBD have different neurophysiological profiles, cognitive impairment (CI) symptomology, and regions of cortical thinning (Gurnani & Gavett, 2017; Kang et al., 2019). Distinct associations between auditory thresholds and vascular and non‐vascular dementia have been reported (Gallacher et al., 2012). In addition, peripheral HL is more prevalent in patients with LBD than in patients with AD (Jung et al., 2021). These findings suggest that the relationship between HL and each type of dementia is underpinned by distinct mechanisms, although the neurocognitive pathologies underscoring this association have not been demonstrated to date.

This study aimed to evaluate the association between age‐related hearing loss (ARHL) and dementia types using brain ^18^F‐fluorodeoxyglucose (FDG) positron emission tomography/computed tomography (PET/CT). Brain FDG‐PET scans are used to evaluate patients with dementia for biomarkers of neurodegenerative diseases based on the differential brain metabolism patterns associated with underlying pathologies (Mosconi et al., 2008). Since the clinical features and metabolic changes in the brain may differ depending on the pathology of CI, the association between ARHL and dementia should be examined separately for each type of pathology. In this study, we analyzed the metabolic changes associated with each CI pathology and the effects of ARHL on brain metabolism.

## 2 METHODS

### 2.1 Participants

**FIGURE 1:**
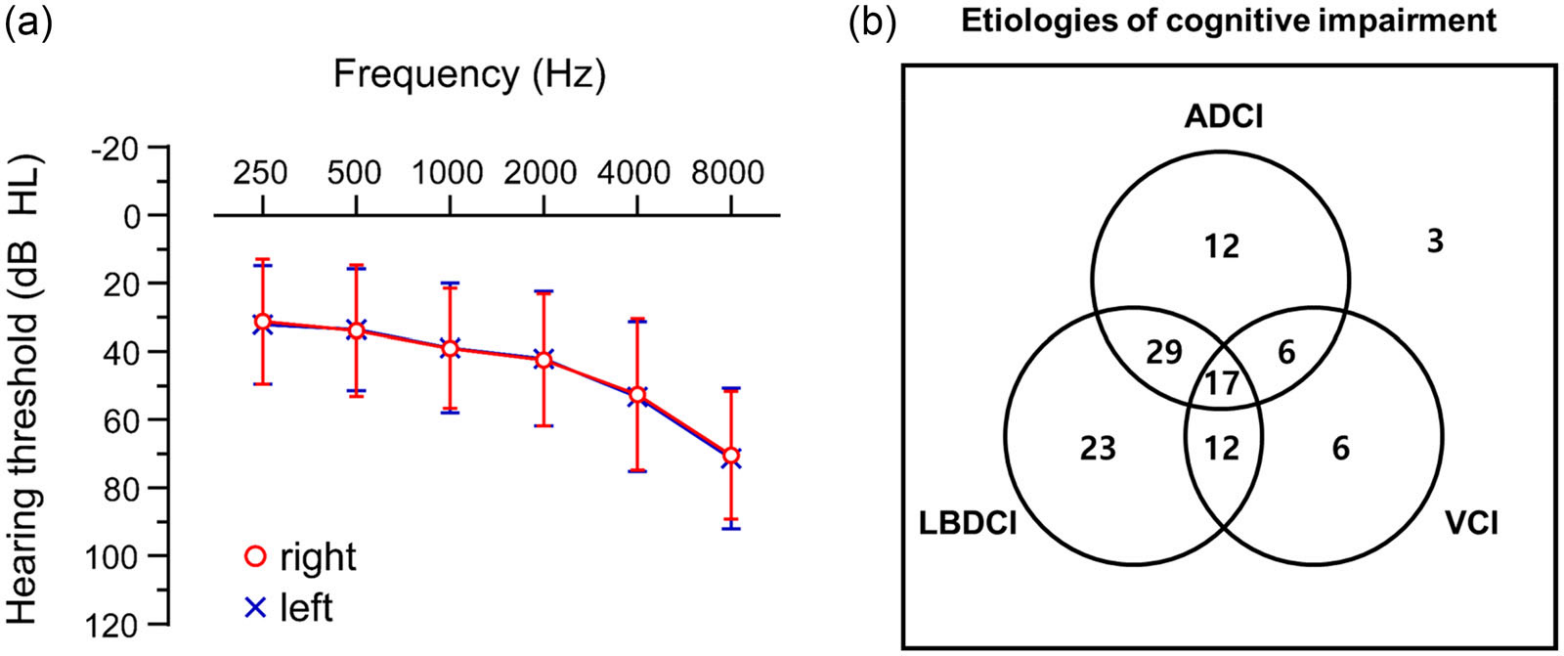
Pure tone audiogram and etiologies of CI in enrolled participants (*n* = 108). (a) Differences in decibels between right and left ear hearing thresholds were less than 5 dB HL for all frequencies. (b) Configurations of causative pathologies of CI. ADCI = Alzheimer's disease‐related CI; dB HL = decibels hearing level; Hz = Hertz; LBDCI = Lewy body disease‐related CI; VCI = vascular CI.

In this prospective cohort study, patients who visited the university‐based memory clinic due to CI and HL were enrolled consecutively from May 2016 to October 2021. All participants underwent hearing evaluation (Figure 1a and Figure S1), neurologic examinations, neuropsychological tests, brain magnetic resonance imaging (MRI), and FDG‐PET. If necessary, additional brain nuclear imaging, including ^18^F‐florbetaben (FBB) PET and ^18^F‐fluorinated N‐3‐fluoropropyl‐2‐beta‐carboxymethoxy‐3‐beta‐(4‐iodophenyl) nortropane (FP‐CIT) PET, was performed to confirm clinical diagnosis. All participants underwent laboratory tests, including complete blood counts, blood chemistry, thyroid function, and apolipoprotein E (APOE) genotyping. A standardized neuropsychological battery (Seoul Neuropsychological Screening Battery) was administered to all participants as previously described (Jung et al., 2021). The Korean version of the Mini‐Mental State Examination (K−MMSE) was performed to evaluate the general cognitive dysfunction. The severity of depressive symptoms was assessed using the Beck Depression Inventory (BDI) and Geriatric Depression Scale (GDS).

The exclusion criteria included (1) HL related to infection, trauma, and congenital causes; (2) conductive HL; and (3) cognitive dysfunction related to normal pressure hydrocephalus, traumatic encephalopathy, large territorial cerebral infarction, acute or subacute cerebral infarction or hemorrhage, frontotemporal lobar degeneration, and atypical Parkinsonism including progressive supranuclear palsy, multiple system atrophy, and corticobasal degeneration.

The Institutional Review Board of our hospital approved the study (IRB No. 4‐2016‐0648) and dictated relevant guidelines and regulations to perform it. This study was conducted following the principles of the Declaration of Helsinki. Written informed consents were obtained from all participants.

### 2.2 Hearing evaluation

Audiology examinations were performed for all participants. The pure tone audiometry (PTA) threshold was assessed from 250 to 8000 Hz in a double‐walled audiometry booth. Average pure tone thresholds of 0.5, 1, 2, and 4 kHz (PTA_4_) were calculated, and a better‐hearing ear PTA threshold was used for analysis. Speech discrimination scores (SDS) were evaluated as a percentage of correctly repeated responses among 50 monosyllabic Korean words at the most comfortable listening level. A higher PTA threshold or lower SDS indicated poorer hearing and vice versa. Hearing impairment was determined if PTA_4_ was more than 25 dB HL. SDS was described in %.

### 2.3 FDG, FBB, and FP‐CIT PET acquisition and interpretation

FDG, FBB, and FP‐CIT PET acquisitions were performed using Discovery 600 (General Electric Healthcare, Milwaukee, MI, USA). Acquisition and reconstruction of FBB and FP‐CIT PET were performed according to the protocols described in our previous study (Y. G. Lee et al., 2018). Sixty minutes following intravenous injection of approximately 4.1 MBq per body weight (kg) of FDG, FDG‐PET images were obtained over 15 min. Spiral computed tomography scans were obtained for attenuation correction according to the protocol: 0.5 s rotation time, 200 mA, 120 kvP, 3.75 mm section thickness, 10.0 mm collimation, and 9.375 mm table feed per rotation. Visual ratings of brain β‐amyloid plaque load (BAPL) score and FP‐CIT PET abnormalities (i.e., nigrostriatal dopaminergic depletion) were performed by an expert in nuclear medicine (M.Y.) and a neurologist with expertise in dementia (B.S.Y.). BAPL scores of 2 and 3 were considered β‐amyloid‐positive. BAPL scores of 1 were considered β‐amyloid‐negative.

### 2.4 MRI acquisition and interpretation

MRI scans were acquired using Philips 3T scanners (Philips Intera: Philips Medical System, Best, The Netherlands). A detailed description of the MRI acquisition methodology was provided in our previous study (Yoo et al., 2018). To measure the severity of subcortical vascular changes on MRI, modified Fazeka's scale for white matter hyperintensities (WMHs) was employed, and manual quantification of lacunes and cerebral microbleeds (CMBs) was performed. Periventricular WMHs were classified as P1 (cap and band < 5 mm), P2 (5 mm ≤ cap or band < 10 mm), or P3 (cap or band ≥10 mm). Deep WMHs were classified as D1 (maximum diameter of deep white matter lesion < 10 mm), D2 (10 mm ≤ lesion < 25 mm), or D3 (≥25 mm). Lacunes were defined as small lesions (≥3 mm and ≤15 mm in diameter) with a high signal on T2‐weighted images, a low signal on T1‐weighted images, and a perilesional halo on fluid‐attenuated inversion recovery images. CMBs were defined as round lesions (<10 mm in diameter) with homogeneous low signal intensities on T2‐weighted gradient recalled‐echo images. WMHs, lacunes, and CMBs were rated by a neurologist (B.S.Y.).

### 2.5 Participant categorization

As AD and LBD are the two most common neurodegenerative causes of dementia and vascular pathology frequently cooccurs with other degenerative pathologies in the elderly (Heidebrink, 2002; Lennox et al., 1989; Perry et al., 1990), study participants were categorized into AD‐related CI (ADCI), Lewy body disease‐related CI (LBDCI), and vascular CI (VCI) groups. ADCI included AD dementia and mild CI (MCI) due to AD. Clinical AD dementia was diagnosed according to the criteria of the National Institute of Neurological and Communicative Disorders and Stroke and Alzheimer's Disease and Related Disorders Associations (McKhann et al., 1984). The diagnosis of MCI due to AD was based on modified Petersen's criteria (Ye et al., 2018). LBDCI included Parkinson's disease (PD) dementia, MCI due to PD, dementia with Lewy bodies (DLB), and MCI due to DLB. PD was diagnosed according to the United Kingdom PD Society Brain Bank diagnostic criteria for PD (Gibb & Lees, 1988). All patients with DLB met the probable DLB criteria (Blanc et al., 2016). VCI was defined as CI associated with focal neurologic signs observed in neurologic examination and vascular brain pathologies confirmed by MRI (D score ≥2 and P score ≥2). In total, 108 participants comprising patients with ADCI, LBDCI, VCI, and other cognitive disorders (Figure 1b) were enrolled. Participants without CI and HL were categorized as the control group (Group 1, *n* = 27).

### 2.6 Statistical parametric mapping (SPM)

Data analysis was performed using statistical parametric mapping (SPM, Wellcome Trust Centre for Neuroimaging, London, UK), which is MATLAB (The MathWorks, Inc, Natick, MA)‐based software (Kiebel & Holmes, 2007). All reconstructed PET images were spatially normalized into the Montreal Neurological Institute template (McGill University, Canada) using transformation module of SPM12 (Ashburner & Friston, 1999). The normalized images were smoothed by convolution with an isotropic Gaussian kernel with 8‐mm FWHM. General linear models after adjustment for age, sex, education level, and medical diseases (hypertension, diabetes, and hyperlipidemia) was used to evaluate the effect of the association between HL and dementia types. We used a voxel height threshold at *p* = .05 (false‐discovery rate correction for multiple comparison). For visualization of the *t* score statistics (SPM[t] map), the significant voxels were projected onto a standard high‐resolution MRI template provided by SPM12, thus allowing anatomical identification (Tables S1–S3).

### 2.7 Statistical analyses

All statistical analyses were conducted using IBM SPSS 23.0. Student's *t*‐test and analysis of variance were used to compare continuous variables. Chi‐square tests were used to compare categorical variables. Post hoc analysis for multiple comparisons was performed using the Tukey's test or Bonferroni correction. Multivariable regression was performed to evaluate brain regions correlated with each pathology type in patients with CI and HL after adjusting for confounding factors (age, sex, education level, hypertension, diabetes, hyperlipidemia, and PTA_4_). For sensitivity analysis, PTA_4_ were unadjusted as appropriate. *p* < 0.05 was considered significant.

## 3 RESULTS

### 3.1 Demographics and clinical characteristics

Clinical and sociological factors of participants with CI and HL are presented in Table 1. The mean participant age was 76.8 ± 6.4 years (*n* = 108). The mean PTA_4_ and SDS were 38.8 ± 16.1 dB HL and 74.1 ± 22.2%, respectively. After segregating individuals with pure CI pathology, a significantly higher prevalence of the APOE4 risk allele was observed in the pure ADCI group than in the pure LBDCI group (overall *p* = .008; pure ADCI vs. pure LBDCI, adjusted *p* = .038). The PTA_4_ was significantly different in the pure CI pathology groups (*p* = .007). Post hoc analysis revealed that the pure ADCI group had better hearing compared to the pure LBDCI (*p* = .009) or the pure VCI (*p* = .041) groups (Table 1 **and** Figure S1). D and P system scores in the pure VCI group were significantly higher than those in the pure ADCI and LBDCI groups.

**TABLE 1:** Demographics and clinical characteristics.

	Total (*n* = 108)	ADCI (*n* = 64)	LBDCI (*n* = 81)	VCI (*n* = 41)	*p* Value	Pure ADCI (*n* = 12)	Pure LBDCI (*n* = 23)	Pure VCI (*n* = 6)	*p* Value
Age (years)	76.8 ± 6.4	76.3 ± 6.6	77.4 ± 6.2	76.6 ± 6.3	.534	74.4 ± 9.0	79.1 ± 6.2	76.8 ± 2.2	.168
Sex (female)	66 (61.1%)	42 (65.6%)	49 (60.5%)	23 (56.1%)	.608	9 (75.0%)	14 (60.9%)	3 (50%)	.554
Education level (years)	8.8 ± 5.2	9.0 ± 5.1	9.2 ± 5.1	8.7 ± 4.7	.879	7.6 ± 5.6	9.5 ± 6.1	6.1 ± 4.0	.377
Duration of CI (years)	2.6 ± 1.9	2.7 ± 2.1	2.7 ± 2.0	2.8 ± 1.7	.993	2.0 ± 1.4	2.5 ± 1.8	2.7 ± 1.9	.666
Vascular risk factor									
HTN	68 (63.0%)	40 (62.5%)	50 (61.7%)	25 (61.0%)	.988	8 (66.7%)	16 (69.6%)	6 (100%)	.340
Diabetes	30 (27.8%)	14 (21.9%)	26 (32.1%)	10 (24.4%)	.356	2 (16.7%)	7 (30.4%)	0 (0%)	.292
Hyperlipidemia	24 (22.2%)	11 (17.2%)	17 (21.0%)	9 (22.0%)	.793	3 (25.0%)	5 (21.7%)	3 (50%)	.429
Smoking	26 (24.1%)	16 (25.0%)	18 (22.2%)	11 (26.8%)	.840	3 (25.0%)	5 (21.7%)	2 (33%)	.881
APOE4 allele	37 (34.6%)	33 (51.6%)	28 (35.0%)	13 (31.7%)	.062	6 (50.0%)	2 (9.1%)	0 (0%)	.008^*^
Hearing level									
PTA_4_	38.8 ± 16.1	33.7 ± 15.2	39.0 ± 17.3	39.7 ± 16.4	.091	30.1 ± 10.1	46.5 ± 17.5	48.5 ± 7.2	**.007**
SDS	74.1 ± 22.2	79.2 ± 19.3	72.9 ± 23.1	73.0 ± 20.5	.167	83.2 ± 16.2	64.3 ± 27.8	66.3 ± 23.0	.099
Neuropsychological status									
K‐MMSE	22.0 ± 4.7	21.3 ± 5.0	21.5 ± 4.7	21.6 ± 4.5	.940	23.4 ± 4.6	22.8 ± 3.9	22.7 ± 5.2	.913
GDS	3.7 ± 0.9	3.7 ± 0.8	3.8 ± 0.8	3.7 ± 0.9	.638	3.1 ± 0.3	3.7 ± 0.9	3.2 ± 1.5	.113
BDI	12.5 ± 8.9	12.2 ± 8.9	12.7 ± 8.8	13.2 ± 9.5	.842	9.2 ± 3.8	13.8 ± 9.2	11.2 ± 5.7	.250
Neurodegeneration in MRI									
D system score	1.7 ± 0.7	1.6 ± 0.7^a^	1.6 ± 0.7^b^	2.3 ± 0.4	**<.001**	1.3 ± 0.3 ^a^	1.4 ± 0.5 ^b^	2.2 ± 0.4	**<.001**
P system score	1.9 ± 0.8	1.9 ± 0.7^a^	1.9 ± 0.8^b^	2.6 ± 0.5	**<.001**	1.5 ± 0.5 ^a^	1.7 ± 0.7 ^b^	3.0 ± 0.0	**<.001**

*Note*: Values are presented as means ± SDs for continuous data or *n* (%) for categorical data. Boldface text indicates statistical significance (*p* value < .05 according to chi‐square or one‐way ANOVA followed by Tukey's correction for multiple comparisons).

^a,b^Significant difference between ADCI and VCI groups(^a^) and LBDCI and VCI groups(^b^), respectively, in post hoc comparisons.

*Significant difference between pure ADCI and pure LBDCI by the Bonferroni corrected post hoc comparison (adjusted *p* = 0.038).

Abbreviations: ADCI, Alzheimer's disease‐related CI; APOE4, apolipoprotein E 4; BDI, Beck Depression Inventory; GDS, Geriatric Depression Scale; HTN, hypertension; K‐MMSE, Korean version of Mini‐Mental State Examination; LBDCI, Lewy body disease‐related CI; MRI, magnetic resonance imaging; PTA_4_, pure tone audiometry threshold; Pure ADCI, pure Alzheimer's disease‐related CI; Pure LBDCI, pure Lewy body disease‐related CI; SD, standard deviation; SDS, speech discrimination scores; VCI, vascular CI.

### 3.2 Group analysis of FDG‐PET

Group analysis was performed to compare the differences in brain metabolism based on the presence of CI and HL severity. Individuals without CI and HL were recruited as a control group (Group 1, *n* = 27). Among participants with CI (*n* = 108), those with minimal‐to‐mild HL (PTA_4_ < 40 dB HL) were categorized as Group 2 (PTA_4_ = 25.4 ± 8.6 dB HL, SDS = 90.5 ± 1.5 %, *n* = 53), and those with moderate‐to‐severe HL (PTA_4_ ≥40 dB HL) were categorized as Group 3 (PTA_4_ = 51.6 ± 10.0 dB HL, SDS = 58.3 ± 20.5 %, *n* = 55). Age and education level were significantly higher in Group 1 than in Group 2 or 3. The prevalence of hypertension, APOE4, and D and P system scores was significantly lower in Group 1 than in Group 2 or 3, whereas K‐MMSE was significantly higher in Group 1 (Table 2).

**TABLE 2:** Intergroup comparison of clinical characteristics.

	Group 1 (*n* = 27)	Group 2 (*n* = 53)	Group 3 (*n* = 55)	*p* Value
Age (years)	72.6 ± 5.2	74.9 ± 6.5	78.6 ± 5.8	**<.001**
Sex (female)	17 (63.0%)	35 (66.0%)	31 (56.4%)	.578
Education level (years)	13.3 ± 4.7	9.0 ± 5.0	8.7 ± 5.4	**<.001**
Duration of CI (years)		2.7 ± 2.0	2.5 ± 1.9	.595
HTN	8 (29.6%)	30 (56.6%)	38 (69.1%)	**.003**
Diabetes	3 (11.1%)	12 (22.6%)	18 (32.7%)	.094
Hyperlipidemia	9 (33.3%)	13 (24.5%)	11 (20.0%)	.418
Smoking	2 (7.4%)	28 (26.7%)	23 (21.1%)	.092
APOE4 allele	5 (18.5%)	24 (45.3%)	13 (24.1%)	**.017**
K‐MMSE	28.5 ± 1.5	21.8 ± 5.1	22.2 ± 4.4	**<.001**
D system score in MRI	1.2 ± 0.3^a^	1.6 ± 0.6	1.7 ± 0.7	**.001**
P system score in MRI	1.2 ± 0.4^a^	1.8 ± 0.7	2.1 ± 0.8	**<.001**

*Note*: Values are presented as means ± SDs for continuous data or *n* (%) for categorical data. Boldface text indicates statistical significance (*p* value < .05 according to chi‐square or one‐way ANOVA followed by Tukey's correction for multiple comparisons).

^a^ Significant differences were found between Group 1 and Groups 2 or 3 in post hoc comparisons.

Abbreviations: APOE4, apolipoprotein E 4; HTN, hypertension; K‐MMSE, Korean version of Mini‐Mental State Examination; SD, standard deviation; MRI, magnetic resonance imaging.

**FIGURE 2:**
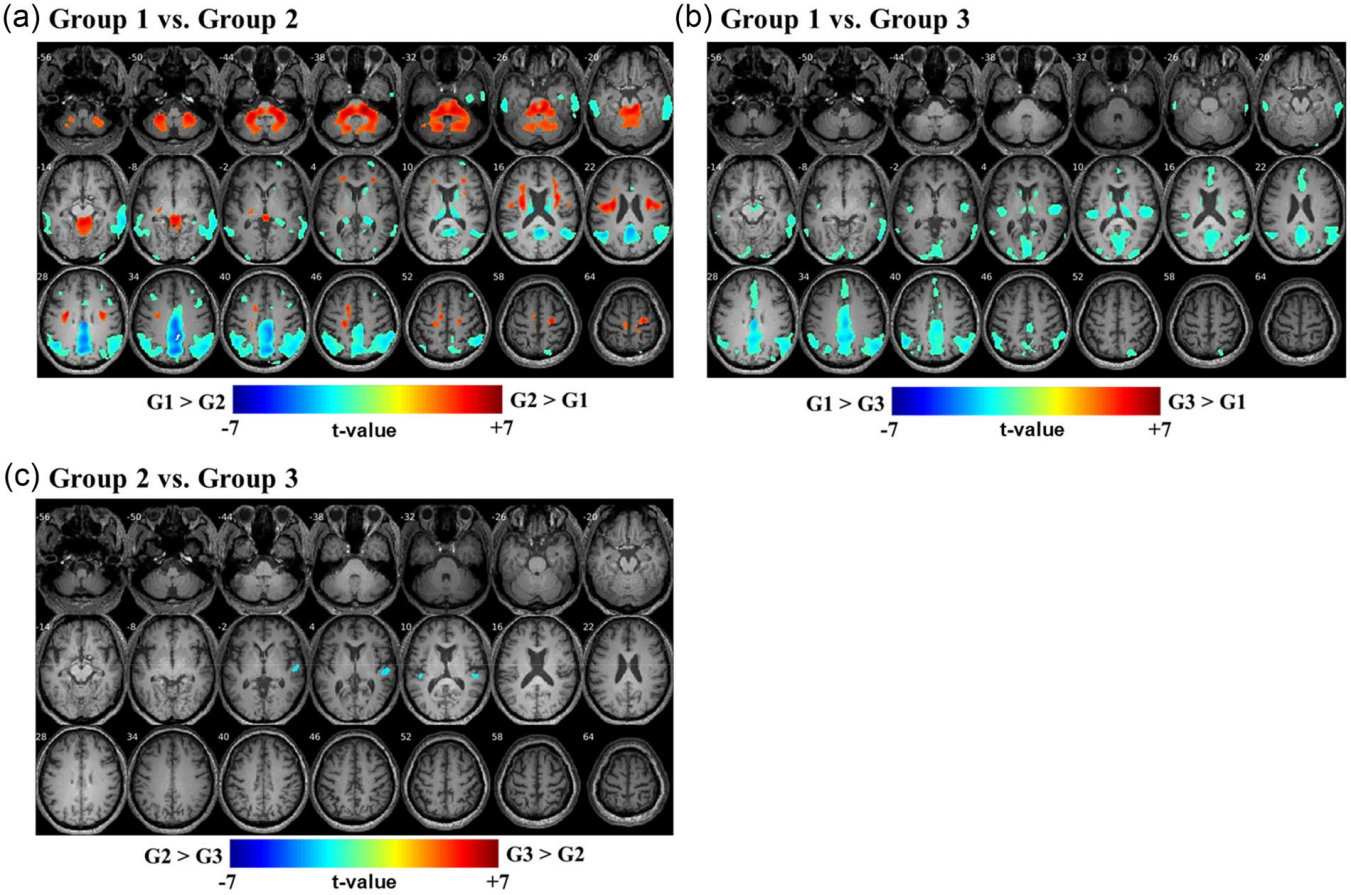
Group analysis of FDG‐PET in patients with cognitive impairment and hearing loss. (a) Compared to Group 1 with normal cognition and hearing (*n* = 27), Group 2 with cognitive impairment and minimal‐to‐mild hearing loss (*n* = 53) exhibited hypometabolism in the bilateral MTG and parietal cortices, PCC, and precuneus; and hypermetabolism in the cerebellum, pons, and midbrain. (b) Group 3 with cognitive impairment and moderate‐to‐severe hearing loss (*n* = 55) exhibited hypometabolism in the ACC, bilateral STG and MTG, bilateral parietal cortices, PCC, and precuneus. (c) Comparison of Group 3 to Group 2 revealed significant hypometabolism in the right STG with a smaller change in the left STG in Group 3. FDG‐PET = ^18^F‐fluorodeoxyglucose (FDG) positron emission tomography (PET); STG = superior temporal gyrus; MTG = middle temporal gyrus; PCC = posterior cingulate cortex; ACC = anterior cingulate cortex; G1 = Group 1; G2 = Group 2; G3 = Group 3.

For group comparisons of FDG uptake on brain PET/CT, we corrected for confounding factors, including age, sex, education level, and hypertension. Compared with Group 1 patients, Group 2 patients exhibited hypometabolism in bilateral temporal and parietal cortices, precuneus, and posterior cingulate cortex and hypermetabolism in the cerebellum, pons, and midbrain (Figure 2a **)**. In CI patients with moderate‐to‐severe HL (Group 3), additional hypometabolism in the anterior cingulate gyrus and bilateral superior temporal gyri (STGs) was observed (Figure 2b). A comparison of Groups 2 and 3 revealed significant hypometabolism in the right STG and a small portion of the left STG in Group 3 (Figure 2c).

### 3.3 Effects of neurocognitive pathologies on auditory pathway and related brain regions

First, we analyzed the association between PTA_4_ (or SDS) and FDG‐PET in patients with all‐cause CI and HL after adjusting for age, sex, education level, and underlying medical diseases. In general, the right STG metabolism was negatively correlated with PTA_4_ (Figure S2a) and positively correlated with SDS (Figure S2b). This indicated that the right STG metabolism decreases as HL progresses in patients with CI and HL.

Then, to investigate the relationship of brain metabolism with HL according to the dementia pathologies, patients were subdivided into ADCI, LBDCI, and VCI groups, and the relationships between HL and each type of CI pathology were evaluated in univariable analyses (Figure S3). In the ADCI and LBDCI groups, the right STG metabolism was negatively correlated with PTA_4_, which was similar with the result with all‐cause CI. We assumed that there might be masked distinct effects of individual dementia pathology on brain metabolism due the frequent cooccurrence of underlying dementia pathologies, as shown in Figure 1b.

**FIGURE 3:**
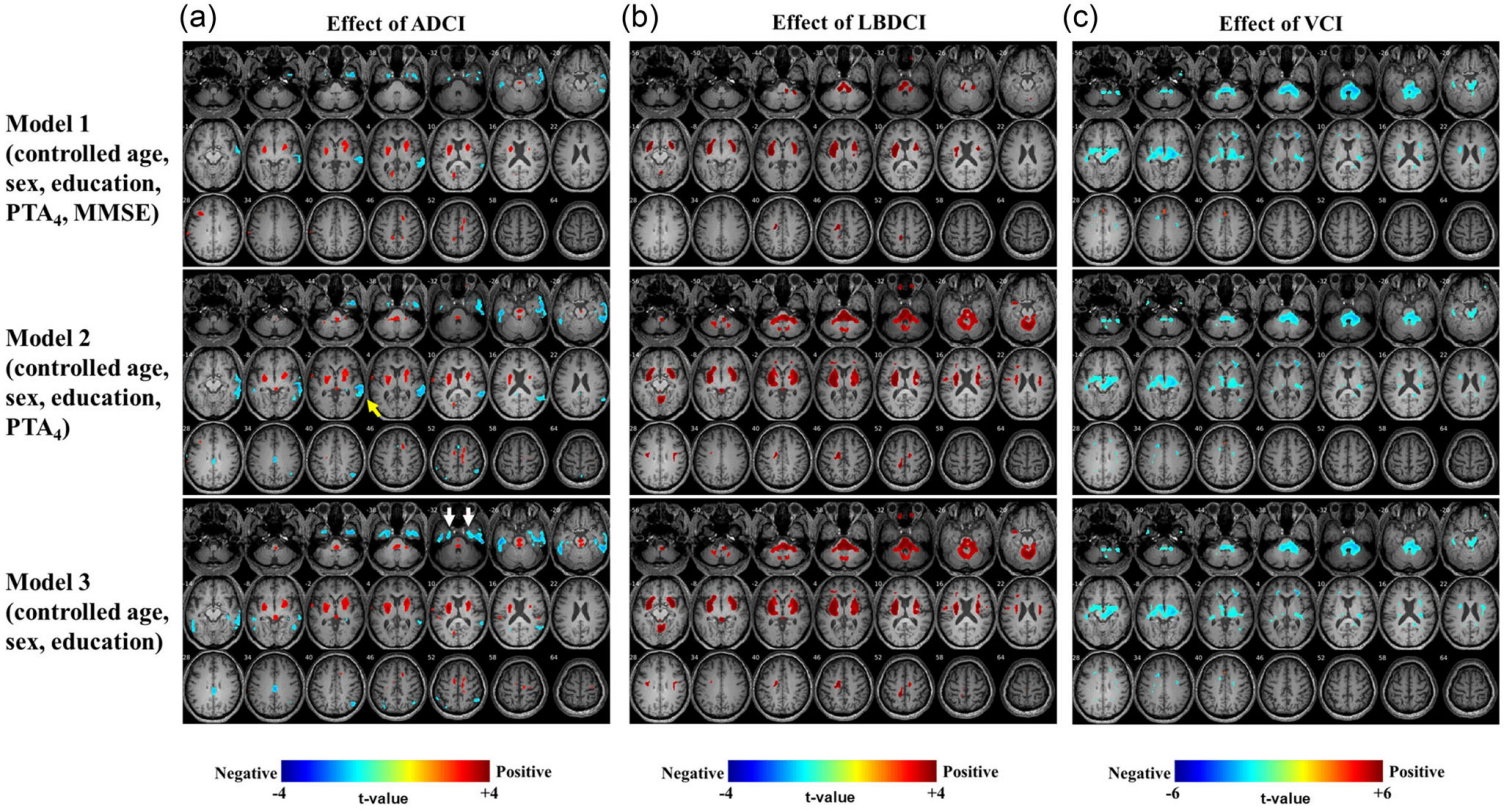
Characteristic effects of each type of CI on brain metabolism (*n* = 108). Model 1 was adjusted for age, sex, education level, hypertension, diabetes, hyperlipidemia, K‐MMSE, and PTA_4_. K‐MMSE was excluded from adjustment in Model 2. PTA_4_ was additionally excluded from adjustment in Model 3. (a) In Model 1, ADCI‐correlated hypometabolism of the right STG, right MTG, and bilateral MTL; and hypermetabolism of bilateral basal ganglia were observed. The hypometabolic areas were more prominent in Model 2, and additional hypermetabolic region in the right parietal area including the angular gyrus was identified **(yellow arrow)**. In Model 3, hypometabolism in the right STG and MTG were not significant as they were in Model 2 and more prominent hypometabolic regions in the bilateral MTL **(white arrows)** was observed. (b) In Model 1, LBDCI patients exhibited in the right cochlear nucleus, pons, and bilateral striatum. The hypermetabolic regions in Model 1 were more prominent in Model 2. Comparing Model 3 to Model 2 revealed no significant differences in brain metabolism. (c) VCI‐correlated hypometabolic changes in the ascending auditory pathway including the pons with bilateral cochlear nucleus, midbrain with bilateral inferior colliculus, and bilateral thalamus with right medial geniculate body were observed in Model 1. No differences in brain metabolism were observed between Models 1, 2, and 3. Model 1 was adjusted for age, sex, education level, underlying disease, PTA_4_, and K‐MMSE scores. K‐MMSE scores were excluded from adjustment in Model 2. PTA_4_ was additionally excluded from adjustment in Model 3. ADCI = Alzheimer's disease‐related CI; K‐MMSE = Korean version of Mini‐Mental State Examination; LBDCI = Lewy body disease‐related CI; MTG = middle temporal gyrus; MTL = medial temporal lobe; PTA_4_ = pure tone audiometry threshold; STG = superior temporal gyrus; VCI = vascular CI.

Therefore, we followed the analyses with multivariable regression for brain metabolism as a function of neurocognitive pathology. All patients with ADCI, LBDCI, and VCI were included in the regression model. In Model 1, we found that ADCI was correlated with hypometabolism of the right STG, right MTG, and bilateral medial temporal lobe (MTL) (Figure 3a, upper panel). In Model 2, the MMSE score was excluded from Model 1 to evaluate the effects of the severity of cognitive function. Model 2 revealed more prominent hypometabolism in the right STG and MTG, and an additional hypometabolic region in the right parietal area, including the angular gyrus, in ADCI (Figure 3a, middle panel). In Model 3, PTA_4_ from Model 2 was removed to assess the effects of HL severity. Model 3 revealed the absence of hypometabolic regions in the right STG and MTG and more prominent hypometabolic regions in the bilateral MTL, indicating that HL severity substantially altered the right STG and MTG, and bilateral MTL metabolism in ADCI (Figure 3a, lower panel).

In LBDCI, significant hypermetabolism in the right cochlear nucleus, pons, and bilateral striatum, including the caudate head, pallidum, and putamen, was observed in the Model 1(Figure 3b, upper panel). When MMSE was removed from the model to assess the effects of cognitive function, the hypermetabolic regions in Model 1 were more prominent and newly included the cerebellum and midbrain in LBDCI (Figure 3b, middle panel). However, no significant changes in brain metabolism in LBDCI were noted when PTA_4_ was removed from Model 2 to assess the effects of HL severity, indicating that HL severity did not impact metabolism in the affected regions (Figure 3b, lower panel). Conversely, significant hypometabolic changes in the pons, midbrain, bilateral thalamus, and scattered focal cortical and subcortical areas were observed in VCI in Model 1 (Figure 3c, upper panel). When MMSE was not included in the model (Model 2), the hypometabolic regions in VCI were not changed in comparison to Model 1 (Figure 3c, middle panel). No significant differences in brain metabolism were noted in VCI when PTA_4_ was removed from Model 2, indicating that HL severity is not a confounding variable (Figure 3c, lower panel). These findings suggest that LBDCI and VCI are associated with the abnormal metabolic changes in the ascending auditory tract including the pons with the cochlear nucleus, midbrain with the inferior colliculus, and thalamus with the right medial geniculate body. The sensitivity analysis with PTA_4_ was consistent with that with SDS (Figure S4).

### 3.4 Effects of HL severity on brain metabolism

**FIGURE 4:**
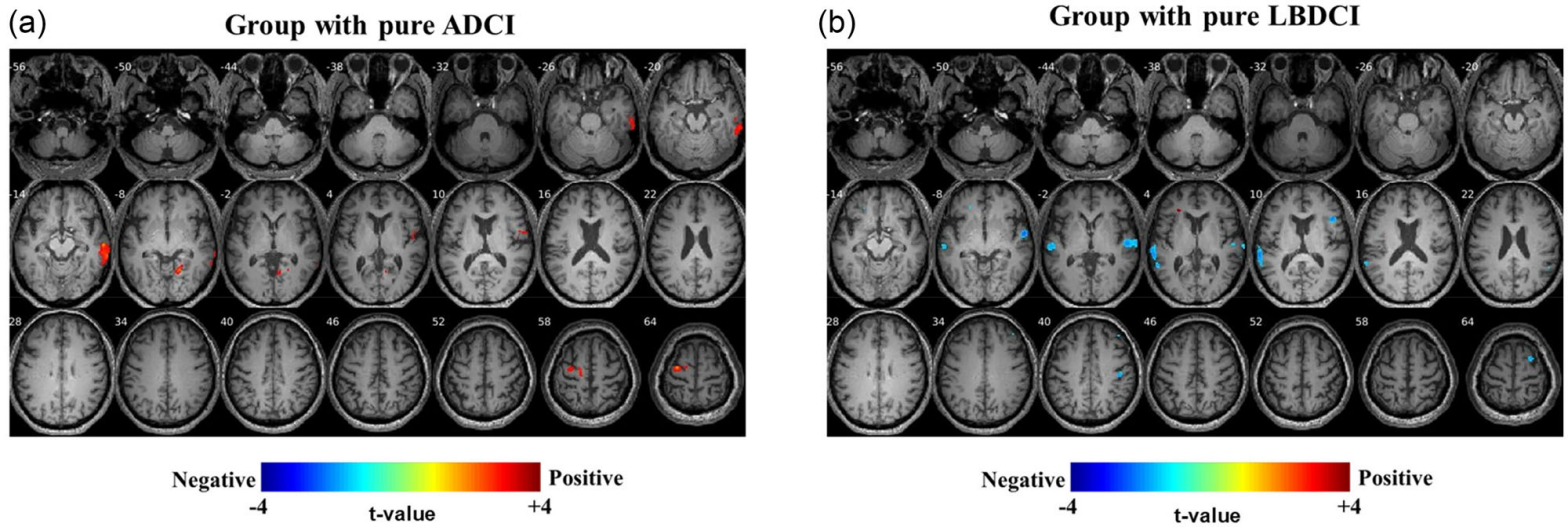
Correlation between severity of hearing loss and brain metabolism in patients with pure ADCI and LBDCI pathology. (a) Right MTG metabolism was positively correlated with PTA_4_ in pure ADCI (*n* = 12). No significant correlation was observed between bilateral STG metabolism and PTA_4_. (b) In pure LBDCI (*n* = 23), bilateral STG metabolism was negatively correlated with PTA_4_. The analysis was adjusted for age, sex, education level, hypertension, diabetes, and hyperlipidemia. MTG = middle temporal gyrus; PTA_4_ = pure tone audiometry threshold; Pure ADCI = pure Alzheimer's dementia‐related CI; Pure LBDCI = pure Lewy body disease‐related CI; STG = superior temporal gyrus.

To identify the effect of HL severity on brain metabolism in each neurocognitive pathology without confounding effects of mixed types of CI, the pure ADCI, LBDCI, and VCI were further segregated and the associations between PTA_4_ and brain metabolism were analyzed in each pure group. The results showed that the right MTG metabolism was positively correlated with PTA_4_ in the pure ADCI group (Figure 4a), indicating that more severe hearing loss increases the right MTG metabolism. FDG‐PET of the pure LBDCI group revealed a negative correlation of bilateral STG metabolism with PTA_4_ (Figure 4b), indicating that more severe hearing loss is associated with the lower metabolism of the bilateral STG corresponding to primary auditory cortex. No statistically significant metabolic lesions were observed for mixed pathologies of ADCI/LBDCI, non‐ADCI/non‐LBDCI, or VCI.

## 4 DISCUSSION

Altered brain metabolism patterns in FDG‐PET are widely employed as diagnostic biomarkers in patients with dementia (Dubois et al., 2014). Hypometabolism of the bilateral temporal and parietal cortices, precuneus, and posterior cingulate cortex are typical patterns observed in patients with ADCI, whereas both hypometabolism of the occipital cortex and temporoparietal association cortices and hypermetabolism of the somatomotor cortex, basal ganglia, and cerebellum are observed in patients with LBDCI (Dubois et al., 2014; McKeith et al., 2017; Mosconi et al., 2008). In VCI, focal cortical, subcortical, deep gray nuclei, and cerebellar hypometabolism is typical (Shivamurthy et al., 2015). The brain areas and changes in metabolism associated with HL include hypometabolism of the STG and auditory tract (J. S. Lee et al., 2003; Speck et al., 2020; Zainul Abidin et al., 2021). Given the significance of HL as a risk factor for dementia, research is needed to investigate the metabolic changes in brain areas associated with both HL and dementia.

A comparison of patients with CI and moderate‐to‐severe HL to those with CI and minimal‐to‐mild HL revealed distinct right STG hypometabolism (Figure 2c), indicating that the right STG is the most affected region by the severity of peripheral HL regardless of the type of CI pathology. This finding is consistent with the previous studies reporting that the STG is the most prominently hypometabolic in patients with HL (J. S. Lee et al., 2003; Okuda et al., 2013). In fact, the STGs receive neuronal projections from the auditory pathway and are involved in speech perception and sound processing (Binder et al., 2000). The right and left STGs are recruited for spectral and temporal resolution, respectively (Zatorre & Belin, 2001). Although STGs are differentially involved in sound processing, HL induces bilateral STG hypometabolism (J. S. Lee et al., 2003). In patients with asymmetric HL, glucose metabolism is lower on the side contralateral to the side with more severe HL (Speck et al., 2020). Notably, hemispheric lateralization of decreased metabolism was observed in the right STG, although all patients had symmetric HL in this cohort (Figure 1a). Although the reason remains elusive, similar right hemispheric lateralization was also reported by a previous study that evaluated hearing impairment and brain volume using MRI (F. R. Lin et al., 2014). Therefore, we consider that the asymmetric hypometabolism in the right STG may be a reliable and prominent biomarker of peripheral HL in patients with CI. However, it is unlikely that the right STG hypometabolism is characteristic of patients with CI and HL, because such hypometabolism is similarly identified in cognitively normal individuals with HL (J. S. Lee et al., 2003; Okuda et al., 2013).

In this study, we compared the brain metabolism in patients with CI and HL and found distinct patterns of metabolic change in ADCI, LBDCI, and VCI. Based on the results of multivariable analyses, hypometabolism in the right MTG was characteristic of patients with ADCI and HL (Figure 3a **, upper panel)**. Indeed, hypometabolism in the right MTG was one of the metabolic biomarkers of AD (Dong et al., 2021; Liang et al., 2008). Dong et al. (2021) reported that the hypometabolism of the right MTG was a biomarker of subjective cognitive decline. Another study by Liang et al. (2008) showed that metabolic changes of the brain in FDG‐PET of AD may be associated with reduced neuronal expression of the nuclear genes encoding subunits of the mitochondrial electron transport chain. Although there is a limitation in that information about hearing level was absent in those previous studies, our findings in Model 3 with additional adjustment of PTA_4_ showed consistent findings of hypometabolism of the right MTG. Moreover, the positive correlation between the metabolism of the right MTG and HL cannot be solely attributed to HL, since this area is not part of the central auditory pathway and does not exhibit metabolic changes as observed in the previous studies (Speck et al., 2020; Zainul Abidin et al., 2021) However, there was some limitation of conducting sensitivity test with two models, as it could not provide a statistically significant difference between the two models. Further study using a larger sample size of pure dementia type with a variable range of hearing loss will guarantee a more precise conclusion.

The MTG is associated with semantic memory and language comprehension (Binder, 2017). The MTG plays a role in sentence comprehension, supported by a widespread network connecting the left posterior MTG with other brain regions (Turken & Dronkers, 2011). Given the functions of the MTG, it can be speculated that the compensation mechanism for cognitive overload from HL may result in increased metabolism in the right MTG in patients with ADCI and HL. Further research is needed to confirm the compensation mechanism.

In addition, we distinctly observed hypometabolic change in the bilateral MTL in the ADCI group. Hypometabolism in the bilateral MTL was more significant when HL was not adjusted, indicating that the metabolism in the bilateral MTL is affected by HL (Figure 3a **, lower panel)**. These findings preliminarily imply that peripheral HL may affect the metabolism of parahippocampal and entorhinal cortex, which is crucial for memory and vulnerable to pathologic changes in AD (Van Hoesen et al., 2000). Based on this speculation, we believe that the relationship between AD and HL favors the cognitive load hypothesis or auditory input deprivation hypothesis rather than the common cause hypothesis (Livingston et al., 2020). Nevertheless, the causal relationships between AD and HL are not fully supported in this cross‐sectional observation study, and warrant further investigation.

Meanwhile, this study revealed that the temporal cortex was unaffected by LBDCI in patients with LBDCI and HL (Figure 3b). Instead, hypermetabolism in the brainstem, pons, midbrain, thalamus, and basal ganglia was noted in LBDCI, which was not as affected by the level of peripheral HL, as seen in Model 2. Based on these findings, we speculate that HL in patients with LBDCI may be attributable to the LBD pathology per se involved in the ascending auditory tract, corresponding to the pons (harboring the cochlear nucleus), midbrain (harboring the inferior colliculus), and thalamus, consequently leading to their abnormal hypermetabolism (Malmierca & Hackett, 2010). Therefore, HL in the LBDCI group could be favorably explained by the common pathology hypothesis. Another study, which reported that neocortical Lewy bodies were associated with hearing impairment after the onset of CI, might underscore our hypothesis (Brenowitz et al., 2020). However, as our study was based on cross‐sectional data, we were unable to establish causal mechanisms. Future prospective observational studies should examine the causal relationship between HL and LBDCI.

VCI is one of the most common causes of dementia after AD, accounting for approximately 15% of reported cases (O'Brien & Thomas, 2015). Although disease classification and diagnostic criteria remain controversial, VCI generally comprises CI caused by either large cortical infarcts or subcortical vascular disease (O'Brien & Thomas, 2015). In the absence of cortical infarcts, risk factors for VCI include advancing age and vascular risk, such as hypertension (Wiesmann et al., 2013). In addition, cochlear degeneration resulting in high frequency HL is attributed to vascular insufficiency caused by hypertension (B. M. Lin et al., 2016). Our findings of hypometabolism in the ascending auditory tract, including the pons, midbrain, and bilateral thalamus, were consistent with the previously reported findings in vascular dementia (Figure 3c) (Kerrouche et al., 2006; Mielke et al., 1994). In addition, we observed that VCI‐correlated hypometabolism in the brainstem and thalamus was unaffected by HL severity. These results suggest that HL and VCI are unlikely to reciprocally drive pathologic burden. Rather, common vascular pathology may cause peripheral HL and VCI concurrently. Vascular risk may deteriorate both the ascending auditory tract and cochlea (causing HL), and cortex (causing VCI).

## 5 CONCLUSION

In conclusion, we identified the brain regions affected by HL in patients with various pathologic types of dementia. Distinct patterns of brain metabolism were observed depending on the dementia pathology. In ADCI, cortical regions, such as the right MTG and bilateral MTL, were the most strongly affected by HL. In LBDCI and VCI, aberrant metabolism in the ascending auditory tract was identified. Our study demonstrates that the association between HL and CI is differentially correlated by various types of dementia pathology. Mechanistic understanding of this relationship will facilitate personalized management of CI and HL.

## AUTHOR CONTRIBUTIONS

**Ji Hyuk Han**: Data curation; investigation; validation; formal analysis; methodology; writing—original draft. **Sangwon Lee**: Methodology; software; data curation; investigation; validation; writing—review and editing. **Seong Hoon Bae**: Methodology; investigation; formal analysis. **Mijin Yun**: Conceptualization; investigation; validation; writing—review and editing; project administration. **Byung Seok Ye**: Conceptualization; investigation; validation; writing—review and editing; project administration. **Jinsei Jung**: Writing—original draft; writing—review and editing; conceptualization; validation; funding acquisition; investigation; formal analysis; project administration.

## CONFLICT OF INTEREST STATEMENT

The authors declare that they have no conflict of interests.

### CONSENT FOR PUBLICATION

All authors have approved the manuscript and agreed with its submission.

### PEER REVIEW

The peer review history for this article is available at https://publons.com/publon/10.1002/brb3.3374.

## Supporting information

Supp Figure Information

Supp Table S1 Information

Supp Table S2 Information

Supp Table S4 Information

## Data Availability

The data that support the findings of this study are available from the corresponding author upon reasonable request.
