# Supplementary material for: Distinct changes in brain metabolism in patients with dementia and hearing loss
Source: Brain Behav. 2024 Jan 6;14(1):e3374. doi: 10.1002/brb3.3374 (PMC10771228; doi:10.1002/brb3.3374)
Supplement: Supplementary file 1 — Supp Figure Information [file BRB3-14-e3374-s002.pdf]

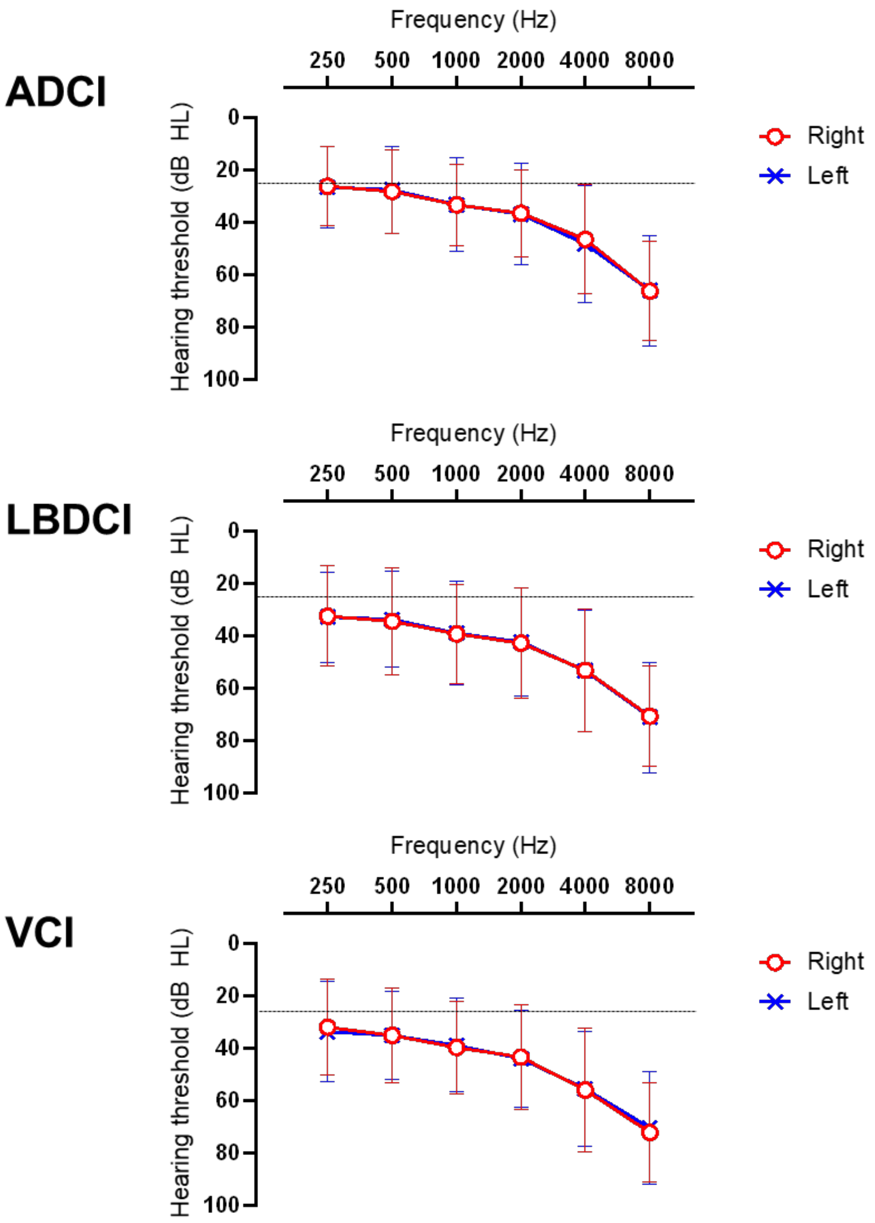

Supplementary Fig. 1 Pure-tone audiogram in the individual dementia pathology Cutoff value for the diagnosis of hearing loss is drawn at 25 dB HL in a dashed line.

Abbreviation: ADCI, Alzheimer's disease-related cognitive impairment; LBDCI, Lewy body disease-related cognitive impairment; VCI, vascular cognitive impairment.; dB HL, decibels hearing level

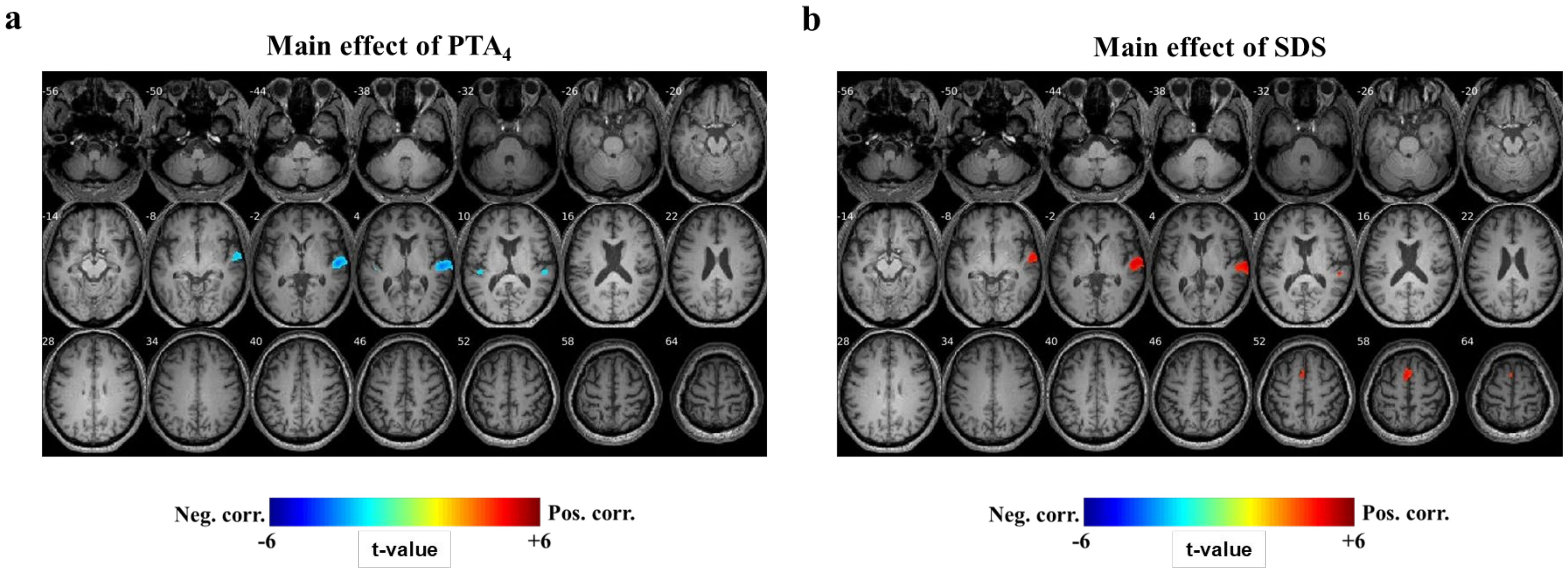

Supplementary Figure 2. Correlation of (a) PTA_4 and (b) SDS with brain metabolism in patients with all-cause cognitive impairment and hearing loss (n = 108). Right STG metabolism was negatively correlated with PTA_4 and positively correlated with SDS. Higher PTA_4 and lower SDS both implied poorer hearing. The analysis was adjusted for age, sex, and education level.

Abbreviation: PTA_4, pure-tone average at 500, 1000, 2000, and 4000 Hz; SDS, speech discrimination score.

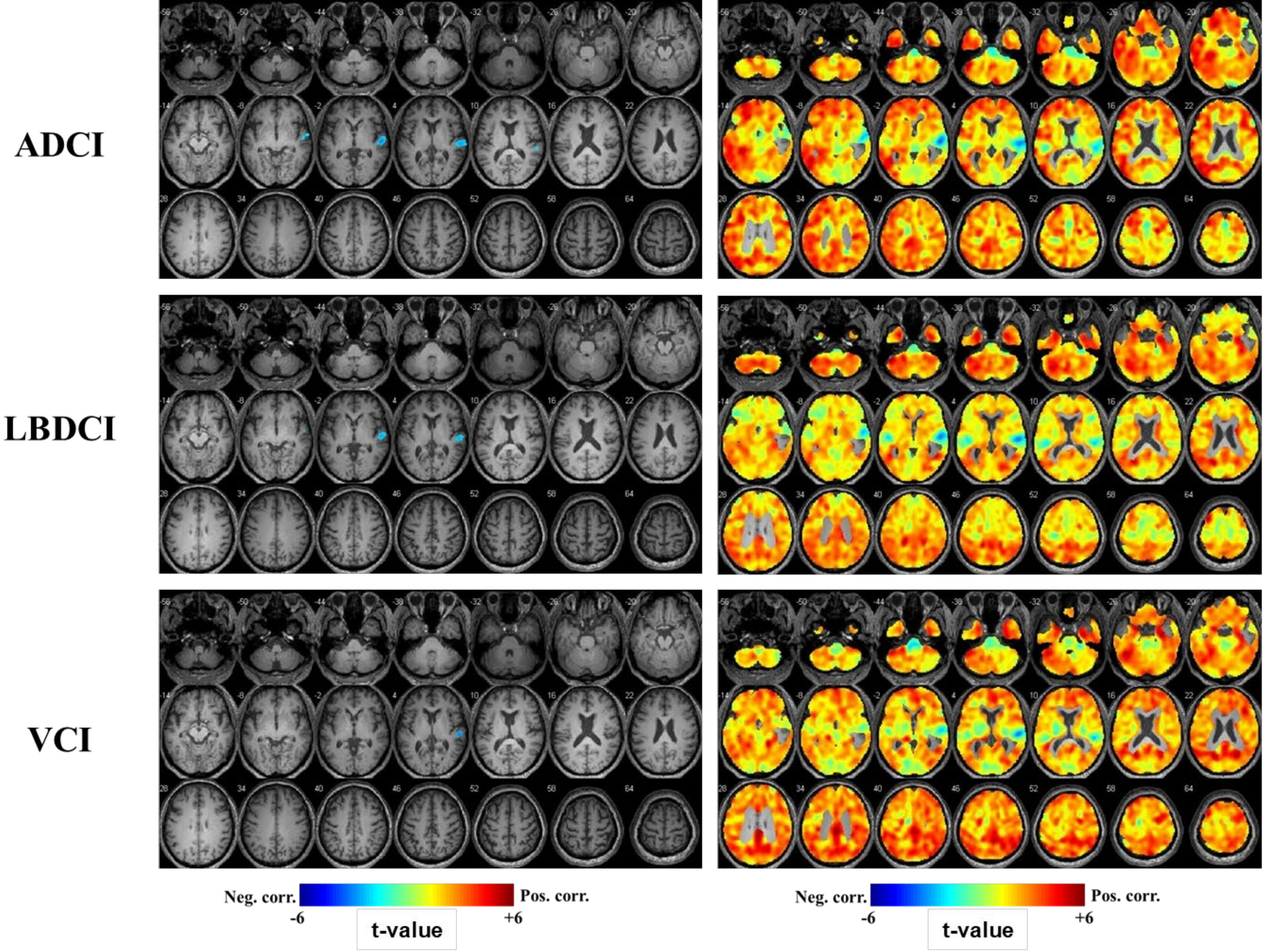

Supplementary Figure 3. Univariable analysis of the correlation between the severity of hearing loss and brain metabolism in each dementia pathology. In the ADCI and LBDCI groups, right STG metabolism was negatively correlated with PTA_4. In the VCI group, metabolic changes in the right STG were not prominent.

ADCI, Alzheimer's disease-related cognitive impairment; LBDCI, Lewy body disease-related cognitive impairment; VCI, vascular cognitive impairment.

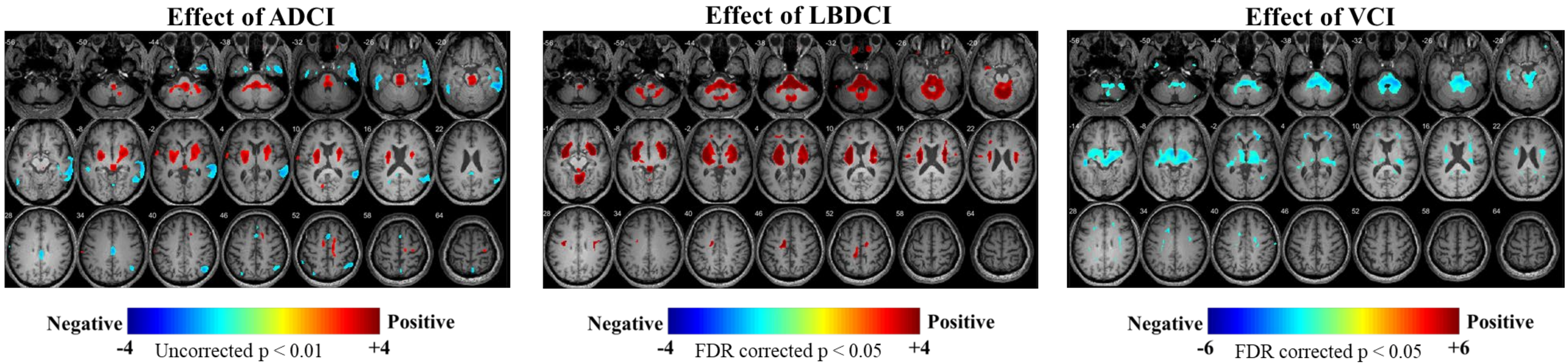

Supplementary Figure 4. Multivariable regression Model 1 with SDS instead of PTA_4. Model 1 (in Fig. 2, upper panel) was adjusted for age, sex, education level, hypertension, diabetes, hyperlipidemia, and SDS (n = 108).

Abbreviation: ADCI, Alzheimer's disease-related cognitive impairment; LBDCI, Lewy body disease-related cognitive impairment; VCI, vascular cognitive impairment; SDS, speech discrimination score.
